# Miniature Dungey-like cycle at Mars

**DOI:** 10.1038/s41467-026-75019-3

**Published:** 2026-07-23

**Authors:** Shaosui Xu, James P. McFadden, David L. Mitchell, Janet G. Luhmann, Jasper S. Halekas, Kathleen G. Hanley, Christian X. Mazelle, Jared R. Espley, Shannon M. Curry

**Affiliations:** 1 Space Sciences Laboratory, University of California, Berkeley, Berkeley, CA USA; 2 Department of Physics and Astronomy, University of Iowa, Iowa City, IA USA; 3 University of Toulouse - CNES - CNRS - IRAP, Toulouse, France; 4 Goddard Space Flight Center, Greenbelt, MD USA; 5 Department of Astrophysical and Planetary Sciences, University of Colorado Boulder, Boulder, CO USA

**Keywords:** Aurora, Magnetospheric physics, Inner planets

## Abstract

With its non-uniform distribution of crustal magnetic fields, Mars exhibits complex and highly variable auroral patterns related to both planetary rotation and solar wind conditions. Using in situ electron, ion, and magnetic field data from the Mars Atmospheric and Volatile EvolutioN (MAVEN) mission, we show that auroral processes associated with these small-scale crustal magnetic fields can be understood in terms of a miniature cycle of magnetic flux and plasma circulations that resemble a miniature version of what occurs at the Earth. However, at Earth, this Dungey cycle, named after its discoverer, operates in the presence of a global intrinsic dipole field with a strength approximately 100 times stronger and spatial scales roughly 20 times larger. From a universal perspective, the current finding adds an entry to the zoo of auroral concepts that enriches our understanding of the diversity of (exo)planetary plasma and our understanding of how planets interact with their space environments.

## Abstract (web summary)

Magnetic flux and plasma circulations around a planet’s dipole field and the associated auroral phenomenon are well studied at many planets. Here, the authors show how it works at Mars’s crustal fields, adding an entry to the collection of auroral concepts.

## Introduction

Auroral emission is a consequence of energetic (greater than 10 s electron volts) particles impacting atmospheric molecules or atoms^1,2^. At Earth, one of the most common auroral forms, discrete aurora, is spatially confined emissions around the magnetic polar regions coinciding with energetic electron precipitation. Auroral electrons at Earth are accelerated up to tens of kilo electron volts (keV)^3^ by processes associated with the reconfiguration of the magnetosphere (the region around a planet dominated by the planet’s magnetic field) in response to incident interplanetary magnetic fields (IMF) with a southward component. These external fields magnetically reconnect with Earth’s dipole-dominated magnetic field on the dayside, changing previously closed field topologies (field lines with both ends connected to the planet) to open field topologies connecting Earth and the solar wind. The newly opened magnetic field lines are convected to the nightside, where they magnetically reconnect again in the magnetotail, changing to closed field lines. This cycle of magnetic field topological change (closed-open-closed), known as the Dungey cycle^4^, controls the circulation of Earth’s magnetospheric and ionospheric plasma flows. As the newly formed closed magnetic field loops relax Earthward, related field-aligned currents (FACs) generate large-scale field-aligned electric fields that accelerate electrons, which impact the atmosphere with a nearly monoenergetic energy spectrum^5^, forming aurorae. These auroras are thus visible signatures of the current system that couples the magnetized solar wind flow to the magnetosphere and ionosphere (the ionized portion of the upper atmosphere).

With a conducting ionosphere and a non-uniform distribution of strong crustal magnetic fields that rotate as part of the planet, Mars presents a somewhat more complex obstacle to the solar wind. In terms of the plasma environment, Mars’s magnetosphere to first order resembles that of Venus, which is dominated by the IMF draping around its ionosphere. However, this relatively simple induced magnetosphere picture is significantly altered from a plasma perspective by its localized intrinsic crustal magnetic fields ^6–9^. Auroral emissions produced by energetic electrons at Mars are observed as both diffuse (widespread and relatively unstructured) and discrete (spatially confined) forms by the Mars Express mission^10–12^, the Mars Atmospheric and Volatile EvolutioN (MAVEN) mission^13,14^, and the Emirates Mars Hope Mission^15^. Among previous findings on auroral phenomena and their source auroral electrons at Mars with MAVEN observations, there is evidence for preferred occurrences over the strongest crustal fields in the southern hemisphere^13,14,16,17^: favoring westward interplanetary magnetic fields (IMF − *B*_*Y*_) and a dusk-dawn local time (LT) asymmetry. The preference for IMF − *B*_*Y*_ is interpreted as a favorable geometry for magnetic reconnection between the crustal magnetic fields and the draped magnetosheath IMFs^17^, a process that changes the topologically closed intrinsic crustal fields to open magnetic field lines, allowing access of external electrons to the atmosphere. These findings hint at the importance of magnetic reconnection in producing these discrete aurorae but provide little information about the auroral electron acceleration processes, as magnetic reconnection itself provides little energization to electrons with a very small Alfven speed (tens of km/s) at Mars^18^.

In this paper, we analyze one case study of the MAVEN observations on 25 February 2017 in detail to reveal a miniature Dungey cycle at Mars. We describe how localized Dungey cycle-like reconnection between the external IMF and crustal magnetic fields at Mars regulates magnetic flux and plasma circulations around the Martian crustal fields. The process by which the Martian discrete auroral electrons are accelerated is shown to be a natural part of this cycle. This study adapts a long-established framework on Earth to the complex crustal magnetospheres at Mars on a spatial scale that is 20 times smaller. This framework includes five elements: magnetic reconnection^19,20^, FACs^11,21^, electron acceleration^11,12,21^, ionospheric flows, and auroral emissions^10,13^. Four of these elements have been studied (mostly individually) in prior research. Our study presents the observations of the fifth element, ionospheric flows, which is a challenging measurement at the limit of the ion instrument’s capabilities. The case study explored in the manuscript not only provides simultaneous observations of three elements (FACs, electron acceleration, and ionospheric flows) but also demonstrates that the directionalities of these three elements are theoretically consistent. Our study provides a framework connecting all five elements to form a coherent physical basis for the study of the Martian aurora that applies to all previous research on this topic. We provide several additional examples to demonstrate the general applicability of this framework.

## Results

### MAVEN observations

**Fig. 1:**
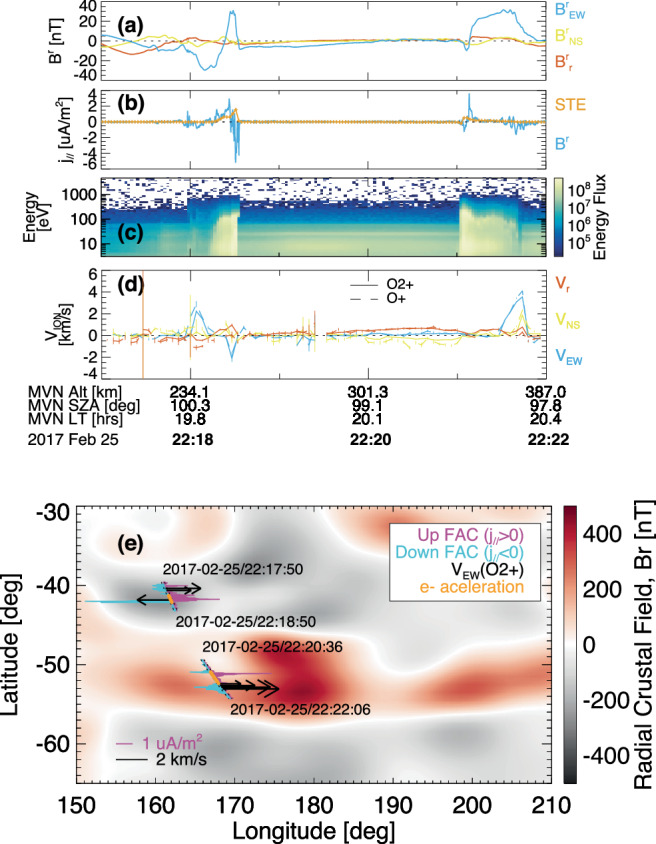
Relevant MAVEN observations of the miniature Dungey-like cycle. Time series of MAVEN observations on 25 February 2017: **a** magnetic perturbation **B**^**r**^ in the local horizontal plane, **b** the derived field-aligned current density from magnetic pertubation ${B}_{EW}^{r}$ (${j}_{//}^{b}$, blue) and superthermal electron fluxes (${j}_{//}^{e}$, orange), **c** superthermal electron energy spectra (differential energy flux in units of eVcm^−2^sr^−1^s^−1^eV^−1^), and **d** the flow velocity of O_2_^+^ (**V**(O_2_^+^)) in the local horizontal plane. **e** The derived FAC (${j}_{//}^{b}$), the east-west component of O_2_^+^ flow velocity (∣*V*_*EW*_(O_2_^+^)∣ > 1 km/s), and electron acceleration observations with ${j}_{//}^{e} > 0.1\,\mu A/{m}^{2}$ as orange dots, overlain on a color map of the modeled radial crustal magnetic field at 250 km altitude^35^. The sign of ${j}_{//}^{b}$ refers to upward (${j}_{//}^{b} > 0$) or downward (${j}_{//}^{b} < 0$) FAC with respect to the local horizontal plane, regardless of whether the local magnetic field is radially upward or downward.

Figure 1 shows MAVEN observations from 22:17 UT (universal time) to 22:22 UT on 25 February 2017 (more detailed MAVEN observations available in Supplementary Figs. S1 and S2). These observations are made at a LT of roughly 20 h, a solar zenith angle of about 100° (near the dusk terminator), and over the strongest crustal fields (part of the orbit tracks shown in Fig. 1e). In Fig. 1c, there are two time periods of electron fluxes significantly elevated from the surrounding time periods, particularly at high energies, and the energies of peak electron fluxes are somewhat time-dispersed, suggesting that these are electrons that have experienced field-aligned acceleration similar to those creating Earth’s discrete auroras^3,21^.

Using an empirical linear relation between electron energy fluxes and the emission brightness^16,22^, these energized/accelerated electrons could produce CO Cameron-band auroral emission (a specific set of ultraviolet emission features of the carbon monoxide molecule) in the Martian upper atmosphere with a limb brightness of about 10 kilo Reighley (kR). This is comparable to some of the brightest auroral observations by MAVEN’s Imaging UltraViolet Spectrograph (IUVS) instrument^13^. In fact, the IUVS instrument observed a CO Cameron-band auroral emission brightness ranging from 0.6 to 1.4 kR at 22:18:37-22:19:30 UT, 25 February 2017, similar to the interval studied, but estimated to occur at [−39.9°, −36.7°] in latitude and [182.5°, 183.0°] in longitude, different from the locations of electron observations. The estimated auroral brightness from auroral electrons at 22:18:20 (closest to the IUVS observations in time) is 4–6 kR, which is a few times the IUVS measurements. It means that the observed electrons in this case study have sufficient energy fluxes to produce observable auroral emissions by IUVS. Their differences are probably due to the time variations in the electron precipitations as well as their different spatial locations.

In the vicinity of the energized electrons, the measured magnetic fields deviate from the local crustal magnetic fields such that the magnetic perturbation **B**^**r**^ from the crustal field (Fig. 1a) is mainly in the local east-west direction. This magnetic perturbation is consistent with a FAC sheet along the east-west direction. With this assumption and the fact that the spacecraft is moving mainly from north to south (Fig. 1e), we derive the FAC density ${j}_{//}^{b}$ from ${B}_{EW}^{r}$, shown as the blue line in Fig. 1b. Note that the sign of ${j}_{//}^{b}$ refers to upward (${j}_{//}^{b} > 0$) or downward (${j}_{//}^{b} < 0$) FAC with respect to the local horizontal plane, regardless of whether the local magnetic field is radially upward or downward. The uncertainty of ${j}_{//}^{b}$ is about 7%, shown in Supplementary Figs. S1f, S3f and S4f. The detailed explanations of the uncertainty calculations for ${j}_{//}^{b}$ and other key parameters are also provided in Supplementary Information. Comparing Fig. 1b, c, accelerated electrons are mostly associated with upward FAC, and the upward FAC is bracketed by two downward FACs for both time periods.

We can also derive the FAC carried by electrons ${j}_{//}^{e}$ based on the observed electron number fluxes, shown as the orange line in Fig. 1b. The uncertainty of ${j}_{//}^{e}$ is about 15%, also shown in Supplementary Figs. S1f, S3f and S4f. The calculated ${j}_{//}^{e}$ has a decent agreement with ${j}_{//}^{b}$ for ${j}_{//}^{b} > 0$ (22:18:15–22:18:28 UT and 22:21:06–22:21:35 UT), supporting the argument that the upward FAC is carried by accelerated downgoing electrons. For times of ${j}_{//}^{b} < 0$ (22:17:55–22:18:15 UT and 22:21:35–22:21:50 UT), ${j}_{//}^{e}$ is near 0, as the downward FAC should be supported by upwelling (denser) ionospheric electrons, and no downward electron acceleration is expected. We note for two particular measurements of ${j}_{//}^{e}$ at approximately 22:18:30 UT and 22:21:05 UT, ${j}_{//}^{e} > 0$ while ${j}_{//}^{b} < 0$, where we expect ${j}_{//}^{e}$ to be around zero. At this time, ${j}_{//}^{b}$ (Fig. 1b, as well as Supplementary Figs. S3 and S4) has rapid variations in its magnitude and signs at a time scale of <1 s, indicating rapid temporal and/or spatial dynamics that are unresolved by the 2-s cadence of the MAVEN electron measurements. In addition to the main case study presented above, we identify six additional events, with their corresponding MAVEN observations shown in Supplementary Figs. S5–S10. In most cases, ${j}_{//}^{e}$ has a decent agreement with ${j}_{//}^{b}$ for ${j}_{//}^{b} > 0$, except for when the change in ${j}_{//}^{b}$ is less than 2 s and too rapid to be resolved by the electron measurement.

Lastly, as shown in Fig. 1d, the calculated bulk flow velocities of ${{{{\rm{O}}}}}_{2}^{+}$ (${{{{\bf{V}}}}}_{{O}_{2}^{+}}$; solid) and O^+^ (${{{{\bf{V}}}}}_{{O}^{+}}$; dashed) in the local plane are very similar. The uncertainties in ${{{{\bf{V}}}}}_{{O}_{2}^{+}}$ and ${{{{\bf{V}}}}}_{{O}^{+}}$ are overplotted as error bars in Fig. 1d, also shown in Supplementary Figs. S1j, S3j and S4j. Both ion flows have a prominent east-west component (*V*_*EW*_ (${{{{\rm{O}}}}}_{2}^{+}$) and *V*_*EW*_(O^+^)) in the vicinity of energized electrons, suggesting the ionospheric bulk flow is in the east-west direction. In particular, there is an east-west flow reversal at 22:18-22:19 UT. More detailed observations and the derivations of **B**^**r**^, ${j}_{//}^{b}$, and ${j}_{//}^{e}$ are provided in the Supplementary Information.

### Miniature Dungey-like cycle at Mars

**Fig. 2:**
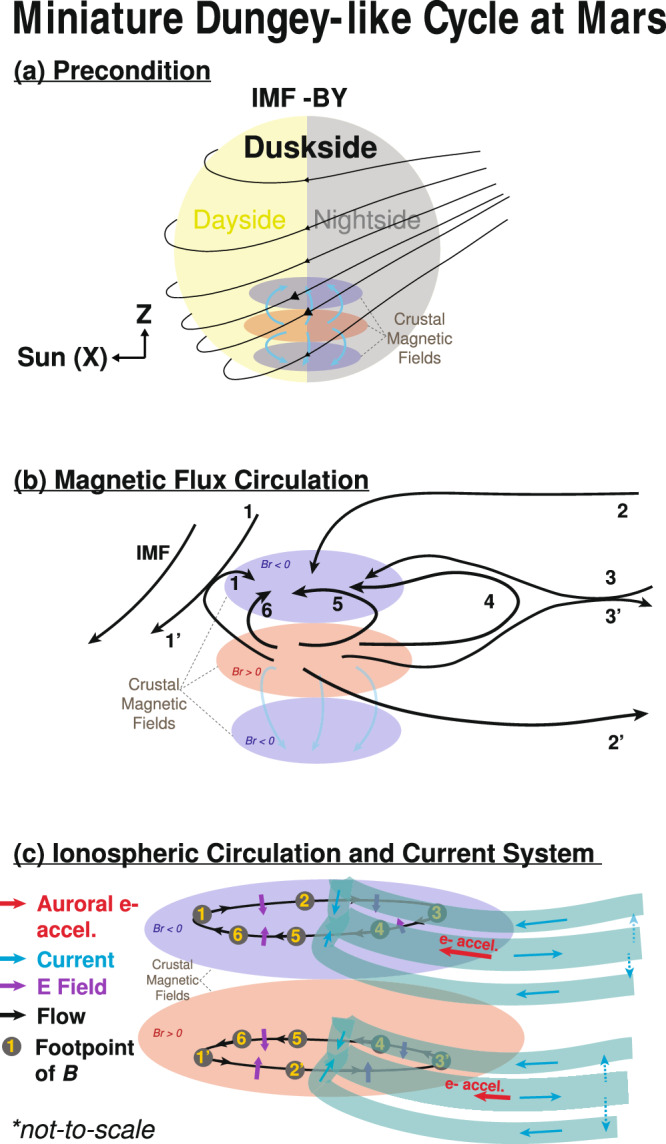
Schematics of the miniature Dungey cycle^4^ at Mars. **a** The precondition for the first magnetic reconnection between the closed crustal magnetic fields and draped interplanetary magnetic field (IMF − *B*_*Y*_) at the dusk terminator. The yellow and gray shaded regions are the dayside and nightside, separately. The blue and red shaded regions are crustal magnetic fields, blue for radially inward magnetic fields (*B*_*r*_ < 0) and red for radially outward magnetic fields (*B*_*r*_ > 0). **b** The magnetic topology change and the magnetic flux circulation of the miniature Dungey cycle. **c** A zoomed-in view of the corresponding ionospheric plasma circulation and current system of the miniature Dungey cycle, with the numbered circles marking the footpoints of the numbered magnetic field lines in (**b**). All the schematics are not-to-scale.

Figure 1e synthesizes these key observations: the FAC density derived from **B**^**r**^ (${j}_{//}^{b}$) shown as magenta (${j}_{//}^{b} > 0$) or cyan (${j}_{//}^{b} < 0$) lines, (auroral) electron observations with ${j}_{//}^{e}$ with a magnitude  > 0.1 μA/m^2^ shown as orange dots, and *V*_*EW*_(O_2_^+^) with a magnitude >1 km/s as the black arrows, all projected onto a crustal field map. Taken together, these can be explained by a miniature Dungey-like cycle of magnetic reconnection and flux and plasma circulation, as illustrated in Fig. 2.

Figure 2a is a sketch of the precondition, where the crustal fields are located near the dusk terminator, the upstream IMF before and after the time of interest being predominantly *B*_*x*_ > 0 and *B*_*y*_ < 0 (the preferred IMF condition for auroral occurrence). The incident draped IMF is tilted southward in the southern hemisphere as a result of momentum exchange between the solar wind and planetary ions, creating a favorable geometry for magnetic reconnection with the local crustal fields, which have a northward horizontal component (the upper arcade)^17^.

Figure 2b illustrates the resulting sequence of magnetic topological reconfiguration consistent with the miniature Dungey-like cycle. It starts with the first magnetic reconnection between the draped IMF and the upper arcade. This reconnection reforms the previously closed field line (1) to two open field lines marked as 2 and 2′. As these open field lines are convected further down the tail by the solar wind flow, the second magnetic reconnection occurs between the oppositely directed open field lines (3 and 3′), generating newly closed field lines (4). These newly closed field lines relax and are then convected Mars-ward (5 and 6), completing the Dungey-like cycle.

**Fig. 3:**
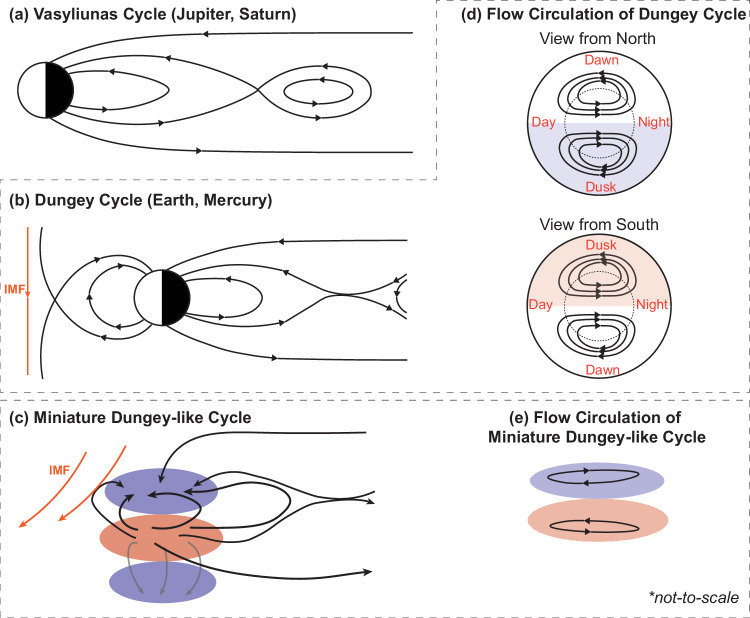
Schematics of three types of plasma and magnetic field cycles. **a** Vasylinuas cycle^30^, operating at giant planets such as Jupiter and Saturn. **b** Dungey cycle, operating at magnetized planets such as Earth and Mercury. **c** Miniature Dungey-like cycle operating at Mars from this study. **d** The flow circulations of the Dungey cycle viewed from North and South, respectively. **e** The flow circulations of the miniature Dungey-like cycle from this study. All the schematics are not-to-scale. IMF is the interplanetary magnetic field.

Associated with this reconnection-driven circulation of magnetic flux, the footpoints of these field lines in the ionosphere are convected by ionospheric plasma flows, as in the case of Earth. As illustrated in Fig. 2c, the flow circulation is most likely clockwise in the upper crustal patch and counterclockwise in the lower crustal patch, so that the Mars-ward convected closed loops (4→5→6) are located towards the center, underneath which are (unopened) closed crustal loops, and open field lines are located outside of closed loops. As these ionospheric flows are driven by the **E** × **B** drift, considering the direction of the local magnetic fields, a set of converging electric fields **E** is needed to generate such flow circulations at both crustal patches. Note that the actual flow circulations do not necessarily extend tens of longitudinal degrees, but could be much more localized than those illustrated, depending on the crustal field pattern. This illustrated miniature Dungey-like cycle resembles half of the Dungey cycle at Earth’s dusk hemisphere, and the two crustal patches where the Mars ionospheric flow circulations take place would be equivalent to the dusk halves of Earth’s northern and southern polar regions, as illustrated in Fig. 3b–d.

The converging electric fields set up pairs of cross-field currents in the ionosphere that close via FACs flowing between the ionosphere and the magnetosphere. FACs flowing away from the ionosphere are supported mainly by precipitating electrons, and if these electrons have insufficient flux to carry the current, magnetic field-aligned large-scale electric fields (double layers) could develop to accelerate electrons toward the planet (resulting in nearly monoenergetic energy spectra) to carry the needed current density. These monoenergetic electrons then produce discrete auroral emissions^11,12,21^.

The MAVEN observations shown here contain the key elements of this process, as highlighted in Fig. 1e. The configuration of downward-upward-downward FACs in both crustal patches in Fig. 2c is consistent with the derived ${j}_{//}^{b}$ shown in Fig. 1b, e. Electron accelerations also mostly coincide with the upward FAC (${j}_{//}^{b} > 0$) observations (Fig. 1b, e), and the FAC carried by these accelerated electrons ${j}_{//}^{e}$ has a reasonable agreement with the upward FAC ${j}_{//}^{b}$. These relationships support the interpretation that electrons are accelerated to carry the needed FACs. Moreover, a prominent east or west (equivalent to tailward or sunward near the dusk terminator, respectively) plasma flow is observed mostly between downward-upward FACs (Fig. 1d, e), consistent with the illustrated flow circulation pattern in Fig. 2c.

**Fig. 4:**
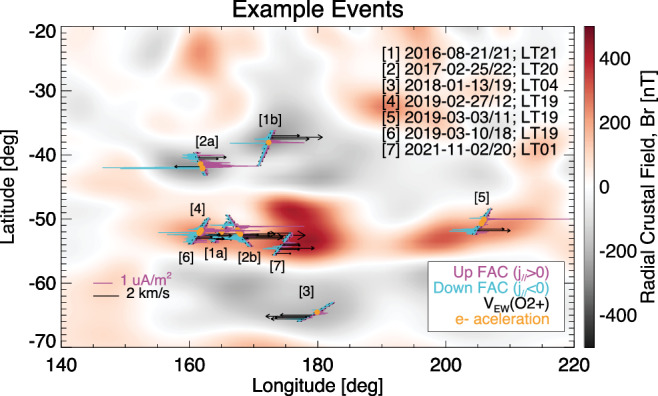
All event examples of MAVEN observations in the geographic coordinates. The derived FAC (${j}_{//}^{b}$), the east-west component of O_2_^+^ flow velocity (∣*V*_*EW*_(O_2_^+^)∣ > 1 km/s), and electron acceleration observations with ${j}_{//}^{e} > 0.1\,\mu A/{m}^{2}$ as orange dots, overlain on a color map of the modeled radial crustal magnetic field at 250 km altitude^35^, for all identified case examples. The date and hour in UT and the local time (LT) of the MAVEN observations for these examples are listed in the lower right corner. The numbers in the brackets are the event numbers, and a and b refer to the two segments of the same event.

In addition to the main case study presented above, Fig. 4 shows the FAC, flow velocity, and electron acceleration observations for all 7 identified case examples overlain on the geographic coordinates. These additional case studies (Events 1, 3–7) have similar signatures to the main case study (Event 2) and occurred at various geographic latitudes and longitudes. Meanwhile, Events 4 and 6 occurred at almost identical geographic locations and LT, despite being observed 11 days apart. Additionally, most of the identified events occurred at latitude [−40°, −55°] during post-dusk, while Event 3 occurred south of these events with the opposite crustal field polarity during pre-dawn (LT 04), consistent with previous findings of the dawn-dusk asymmetry in auroral observations^14^. The occurrence of these events at different crustal fields or at the same location but at different times suggests that this miniature Dungey-like cycle operates regularly at these miniature magnetospheres.

## Discussion

The spatial and temporal scales of this miniature Dungey-like cycle involving the Martian crustal field magnetospheres compared to those at Earth are of interest from a comparative planetology standpoint. We use values from the main case study for this discussion. In the Earth scenario, the second/tail reconnection occurs at about 15 Earth radii down the magnetotail. Scaling this to the dimensions of Mars’s crustal field arcades (an Earth dipole (pole-to-pole) size of 2 Earth radii vs a Mars crustal field dipole size of 1000 km), one would expect to find the second reconnection at Mars to be a few Mars radii downstream (roughly 7500 km). Assuming open field lines are convected downstream at a shocked solar wind flow speed of ~100 km/s, this leads to a time scale of order 1 min for the Mars Dungey-like cycle, compared to order 1 h for Earth’s, but comparable to the cycle period at Mercury^23^. The ionospheric flow at Mars is about 2 km/s, similar to Earth values. With a local magnetic field strength of about 270 nT, this places the cross-field electric field that drives the flow to be ∣**E**_**⊥**_∣ = 0.5 mV/m. The current system from the case study spans about 3° in latitude (about 200 km), and the potential between each set of the downward-upward FACs (a scale length of 100 km) would be roughly 50 V. This potential is about 3 orders of magnitude smaller than Earth’s quiet-time cross-polar-cap potential (tens of kV), with a scale length smaller by one order of magnitude and a local magnetic field strength smaller by two orders of magnitude. With a cycle of approximately 2.5 min (a half cycle of approximately 75 s), we can infer a longitudinal span of the Mars ionospheric flow circulation to be 300 km, or about 7° at −45° latitude.

We can discuss the power dissipation of this cycle in the ionosphere. We first estimate the Pedersen conductivity (*σ*_*P*_) at the footpoints of the field lines to be 5 × 10^−3^ S/m (peak *σ*_*P*_ located at approximately *z* = 180 km altitude), using a neutral CO_2_ density of 10^8^ cm^−3^ and an O_2_^+^ density of 10^4^ cm^−3^ based on the MAVEN observations for this day, and a magnetic strength of 500 nT (scaled from a line dipole located at the surface, ∣**B**∣ proportional to 1/*z*^2^). Assuming the cross-field electric field to be the same at the footpoints (**E**_**⊥**_ = 0.5 mV/m), the cross-field current density **J**_**⊥**_ is 2.5 μA/m^2^, calculated as

1 $${{{{\bf{J}}}}}_{{{\perp }}}={\sigma }_{P}{{{{\bf{E}}}}}_{{{\perp }}},$$

consistent with the observed FAC density. This leads to a local dissipation rate **J**_**⊥**_ ⋅ **E**_**⊥**_ of 10^−9^ W/m^3^ and a total dissipation rate *P* of 10^6^ W, calculated as

2 $$P={{{{\bf{J}}}}}_{{{\perp }}}\cdot {{{{\bf{E}}}}}_{{{\perp }}}V,$$

assuming a dissipation volume *V* of 20 km in altitude (2 neutral scale heights), 200 km in latitude, and 300 km in longitude.

It is also worth noting that the Emirates Ultraviolet Spectrometer (EMUS)^24^ onboard the Emirates Hope Mars Mission (EMM)^25^ has observed more diverse auroral forms than the MAVEN/IUVS observations at not only strong crustal fields but also at weakly magnetized regions^15,26–28^. The EMM/EMUS instrument has a much higher sensitivity than the MAVEN/IUVS instrument. Consequently, EMUS can detect much weaker auroral emissions that result from the precipitation of solar wind electrons^15^ and ionospheric photoelectrons^26,28^, and not just from energized or accelerated electrons that have much higher energy fluxes. In comparison, the auroral detections by the IUVS instrument are mostly limited to moderate/strong crustal field regions, suggesting that these emissions are triggered by electrons likely energized (with more intense fluxes) by processes associated with strong crustal fields. Another piece of supporting evidence is that the occurrence rate of EMUS aurorae is around 10–30% (except for permanently closed crustal field regions)^26^, comparable to the occurrence rates of magnetic field lines allowing for solar wind electrons or photoelectrons to precipitate onto the nightside atmosphere^29^. Meanwhile, the occurrence rate of the IUVS aurora is on the order of 0.1% to a few percent^13^. It suggests that aurorae detected by EMUS are sourced by common electron populations, while aurorae detected by IUVS are sourced by comparatively rare electron populations, such as those accelerated by field-aligned potentials.

The results of this study address the role of discrete auroras in the coupling of the solar wind, magnetosphere, and ionosphere at a planetary body, and in particular, the fundamental contributions that auroral electron acceleration, magnetic reconnection, and flux circulation make to the related current closure. At Earth, the circulation is driven by the interaction between the southward IMF and the global dipolar planetary field, i.e., the Dungey cycle^4^. It is worth mentioning here that at the giant planets Saturn and Jupiter, which also have global dipole fields, there is a more dominant circulation process, known as the Vasyliunas cycle^30^, related to the presence of internal sources of magnetospheric plasma that make corotational dynamics dominate magnetotail reconnection, as illustrated in Fig. 3a. It is therefore noteworthy that, in the Martian miniature magnetospheres, our study reveals a more Earth-like physical picture, including the role of auroral electrons in the coupling current closure. With the exception of its scale, described above, the Martian case shares several similarities to Earth’s Dungey cycle, although only half of the crustal field “dipole” is exposed to the solar wind, with the other half buried beneath the surface. Furthermore, this cycle should regularly operate in a similar fashion over the planet’s many crustal magnetic field sources as Mars rotates and as interplanetary conditions vary. The miniature Dungey-like cycle thus represents a third type of cycle involving planetary magnetic flux and plasma coupling with the solar wind, and related discrete aurorae, that operates in the solar system. This both extends our understanding of the diversity of planetary plasma environments here and elsewhere, and enhances our ability to use remote sensing of aurorae as a means of gaining insight regarding planetary magnetic fields.

## Methods

### Observations

This study uses data from the MAVEN mission, including superthermal electron observations from the Solar Wind Electron Analyzer (SWEA) instrument^31^, magnetic field measurements from the Magnetometer (MAG) instrument^32^, and ion observations from the suprathermal and thermal ion composition (STATIC) instrument^33^. The SWEA instrument provides electron measurements at an energy range of 3–4600 eV with 64 energy steps with a Δ*E*/*E* = 16.7%, with a field of view of 360° × 120° at an angular resolution of 22.5° × 7°, and with a measurement cadence of 2–4 s. The STATIC instrument measures ions over an energy range of 0.1 eV to 30 keV at an energy resolution of Δ*E*/*E* = 16%, with a field view of approximately 360° × 90° at an angular resolution of 22. 5° × 6°, with a measurement cadence of 4 s, and with a time-of-flight system capable of distinguishing major ions at Mars at mass-over-charge (m/q) of 1 (*H*^+^), 2 (${H}_{2}^{+},H{e}^{++ },...$), 16 (O^+^), 32 (${O}_{2}^{+}$), and 44 ($C{O}_{2}^{+}$). The magnetometer measures the magnetic vector with an accuracy of about 0.1 nT over a dynamic range of ±65536 nT at a cadence of 1/32 s.

## Supplementary information

Supplementary Information

Transparent Peer Review file

## Source data

Source data

## Data Availability

MAVEN data are publicly available through the Planetary Data System (https://pds-ppi.igpp.ucla.edu/mission/MAVEN). The following version numbers, where applicable, were used: MAVEN-STATIC: v02; MAVEN-MAG: v01_r01; MAVEN- SWEA: v05_r02. Upstream drivers for the MAVEN mission are publicly available at https://homepage.physics.uiowa.edu/j̃halekas/drivers/. The source data files for the figures in this current study are provided at 10.5281/zenodo.19476134. This file contains a README file that describes the formatting of the source data. Source data are provided with this paper.

## Code availability

Data access and processing were done using SPEDAS V3.1 (http://spedas.org/wiki/index.php?title=Downloads_and_Installation)^34^. The crustal field model can be retrieved using routines in “/project/maven/models/” within SPEDAS. Code written to perform the detailed analysis and to produce the figures in this manuscript can be provided upon request, but with no guarantee that it will work on individual user systems and setups.
