# Supplementary material for: Miniature Dungey-like cycle at Mars
Source: Nat Commun. 2026 Jul 23;17:6129. doi: 10.1038/s41467-026-75019-3 (PMC13396348; doi:10.1038/s41467-026-75019-3)
Supplement: Supplementary file 1 — Supplementary Information [file 41467_2026_75019_MOESM1_ESM.pdf]

Supplementary Information For "Miniature Dungey-like cycle at Mars"

Shaosui Xu[1*], James P. McFadden[1], David L. Mitchell[1], Janet G. Luhmann[1], Jasper S. Halekas[2], Kathleen G. Hanley[1], Christian X. Mazelle[3], Jared R. Espley[4], Shannon M. Curry[5]

[1]Space Sciences Laboratory, University of California Berkeley, 7 Gauss Way, Berkeley, 94720, California, USA.
[2]Department of Physics and Astronomy, University of Iowa, Iowa City, Iowa, USA.
[3]University of Toulouse - CNES - CNRS - IRAP, Toulouse, France.
[4]Goddard Space Flight Center, Greenbelt, Maryland,USA.
[5]Department of Astrophysical and Planetary Sciences, University of Colorado Boulder, 2000 Colorado Ave, Boulder, 80305, Colorado, USA.

*Corresponding author, Email: shaosui.xu@ssl.berkeley.edu;

Contents of this file

Supplementary Fig. S1
Supplementary Fig. S2
Supplementary Fig. S3
Supplementary Fig. S4
Supplementary Fig. S5
Supplementary Fig. S6
Supplementary Fig. S7
Supplementary Fig. S8
Supplementary Fig. S9
Supplementary Fig. S10

Introduction

This document encloses supplementary figures for the manuscript "Miniature Dungey-like cycle at Mars."

More detailed MAVEN observations. Supplementary Fig. S1 shows more detailed information for the case study (Event 2) in the main article. Particularly, the calculation of $\mathbf{B^r}$ relies on the estimation of the unperturbed intrinsic crustal magnetic fields $\mathbf{B_c}$. As this study focuses on some of the strongest crustal fields at Mars that are well captured by crustal field models, we use the adjusted modeled crustal field [1] as the baseline to be subtracted from the observed magnetic fields $\mathbf{B_{obs}}$ to obtain $\mathbf{B^r}$. $\mathbf{B_{obs}}$ is the 32-Hz magnetic field measurements, which are then smoothed over 8 data points (0.25 s) to remove shorter time-scale variations.

Calculation of magnetic perturbation $\mathbf{B^r}$. To capture small-scale magnetic perturbations caused by FACs, adjustments to the modeled crustal field are needed to remove the possible (spatially) large-scale induced magnetic fields from the interaction between Mars and the solar wind. We first obtain linear fits between the observed and modeled magnetic fields for each vector component within 5 minutes bracketing the time of interest, which are shown in Supplementary Fig. S2. The fitting results have a slope of 0.9–1 and mainly an offset, which implies there is a large-scale (almost) uniform background added to the crustal fields, likely rising from the Sun-Mars interaction.

We then recalculate and adjust the "modeled" fields ($\mathbf{B_c'}$, dashed lines in Supplementary Fig. S1a) using these linear fits to capture the smooth variation of the observed magnetic fields, which include both the crustal fields and the large-scale induced magnetic fields. We illustrate the comparison of the observed and adjusted modeled field component in the east-west direction in Supplementary Fig. S1b to better highlight the main magnetic perturbation. The difference between the observed and adjusted "modeled" fields $\mathbf{B^r} = \mathbf{B_{obs}} - \mathbf{B_c'}$ (Supplementary Fig. S1c) reflects the perturbations from currents. Indeed, the calculated $\mathbf{B^r}$ is mostly zero except in the vicinity of accelerated electrons.

Calculation of the current density from magnetic perturbation $j^b_{//}$. By assuming a field-aligned current sheet along the east-west direction and the spacecraft moving mainly from north to south, we can derive the current density mainly in the

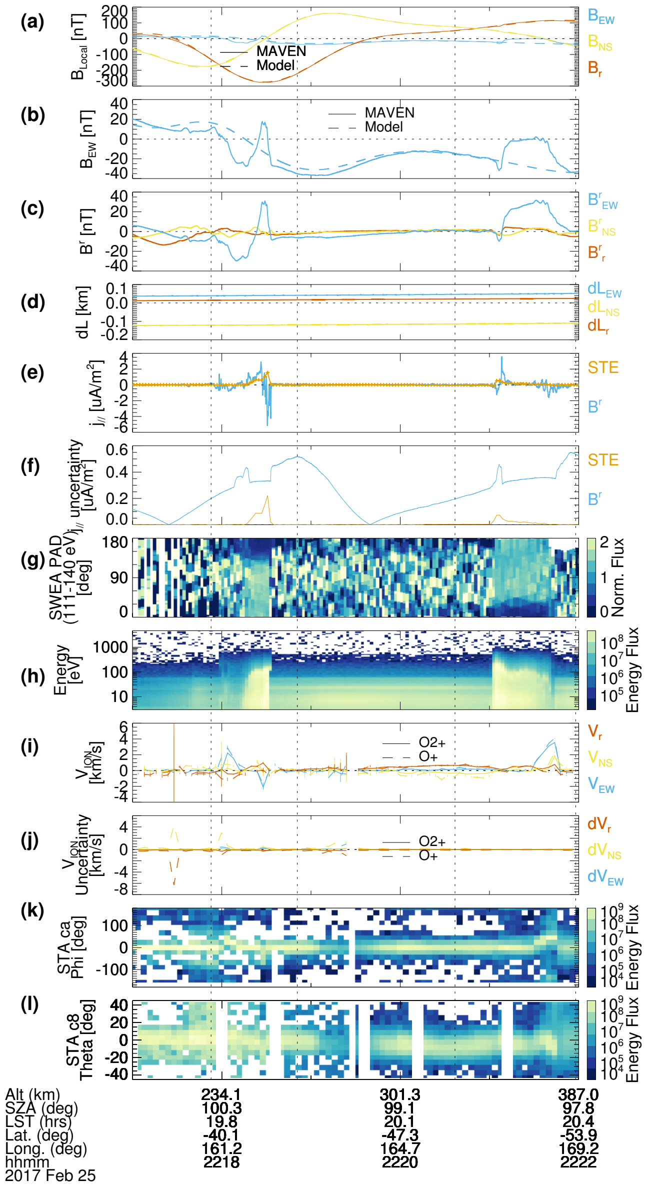

Supplementary Fig. S1 More detailed MAVEN observations on 25 February 2017. Time series of (a) measured (solid) and modeled (dashed) magnetic field vectors in the local horizontal plane, (b) measured (solid) and adjusted modeled (dashed) magnetic field in the east-west direction, (c) magnetic residual $\mathbf{B^r}$ in the local horizontal plane, (d) spacecraft movement $\mathbf{dL}$ in the local horizontal plane, (e) field-aligned current density $j_{//}$ estimated from magnetic pertubation B^r_{EW} (blue) and superthermal electron fluxes (orange), (f) the uncertainty in the calculated $j^b_{//}$ (blue) and $j^e_{//}$ (orange), (g) normalized electron pitch angle distributions (unitless) at 111-141 eV by the averaged energy flux of each measurement, (h) superthermal electron energy spectra, (i) the flow velocity of $O_2{}^+$ ($\mathbf{V}_{O_2+}$; solid) and O^+ ($\mathbf{V}_{O+}$; dashed), (j) the uncertainty in $\mathbf{V}_{O_2+}$ (solid) and $\mathbf{V}_{O+}$ (dashed), the angular distribution of ions in the instrument (k) ϕ (anodes) and (l) θ (deflection) directions. Panels (h), (k), and (l) show differential energy flux in units of $eV\ cm^{-2} sr^{-1} s^{-1} eV^{-1}$.

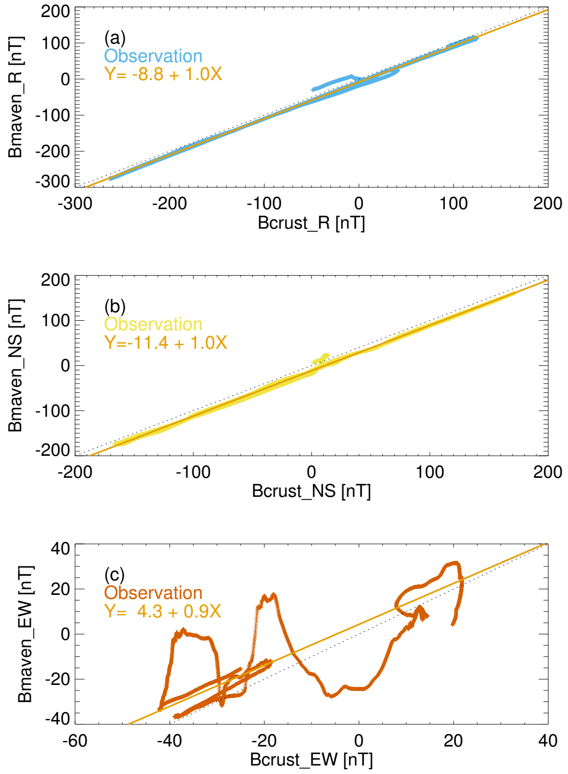

Supplementary Fig. S2 Linear fits between the observed magnetic fields and the modeled crustal fields (Event 2). Three panels show the observed magnetic fields vs the modeled crustal fields [1] as crosses for the (a) radial, (b) north-south, and (c) east-west components, respectively, all transformed to the local plane. The orange line in each panel is the linear fit between the observed and modeled magnetic fields, with the fit function listed in the upper left corner.

radial direction j_r:

$$j_r = \frac{1}{\mu_0}\frac{\Delta B^r_{EW}}{dL_{NS}} \tag{1}$$

where $\Delta B^r_{EW} = B^r_{EW}[t] - B^r_{EW}[t-1]$ is the difference in B^r_{EW} between two time steps and dL_{NS}=$L_{NS}[t] - L_{NS}[t-1]$ is the spacecraft motion in the north-south direction L_{NS} between two time steps. Then the field-aligned component of the current $j^b_{//}$, FAC, is calculated as

$$j^b_{//} = j_r \cdot |b_r| \tag{2}$$

where $|b_r|$ is the absolute value of the radial component of the unit vector of the measured magnetic field. The absolute value of b_r is used so that the sign of FAC refers to upward ($j^b_{//} > 0$) or downward ($j^b_{//} < 0$) FAC with respect to the local plane, rather than the local magnetic field. The calculated $j^b_{//}$ is shown as the blue line in Supplementary Fig. S1e with its uncertainty shown as the blue line in Supplementary Fig. S1f. The uncertainty in $j^b_{//}$ is calculated using the uncertainty in the measured

magnetic field ΔB for each magnetic field component, where $\Delta B/|B| = 0.5\%$ for $|B| > 20$ nT and $\Delta B = 0.1$ nT for $|B| < 20$ nT.

Calculation of the current density from electrons $j^e_{//}$. The electron current density $j^e_{//}$ is calculated from MAVEN SWEA measurements as:

$$j^e_{//} = -e \iint f[E, \theta] \sin\theta \cos\theta 2\pi \, dE \, d\theta \tag{3}$$

where $f[E, \theta]$ is the electron differential number flux in the unit of $cm^{-2}s^{-1}sr^{-1}eV^{-1}$, E is electron energy, and e is the elementary charge. θ is the adjusted pitch angle, the angle between electron velocity and $-|b_r|$, so that $\theta < 90°$ refers to downgoing electrons and $\theta > 90°$ upgoing electrons. This is to have the sign of $j^e_{//}$ also refer to upward or downward FAC to be consistent with $j^b_{//}$. The integration of energy excludes electrons below 20 eV, as these electrons may include secondary electrons produced by precipitating electrons impacting the atmosphere and interior surfaces of the electron instrument. To accurately remove secondary electrons produced within the instrument at different angular bins is very difficult. The calculated $j^e_{//}$ is shown as the orange line in Supplementary Fig. S1e with its uncertainty shown as the orange line in Supplementary Fig. S1f. The uncertainty in $j^e_{//}$ is calculated using the uncertainty in the measured electron flux, comprised of the counting statistic uncertainty, 10% uncertainty in the angular calibration, and 10% uncertainty in the absolute flux (mostly dominated by the last two terms).

Calculation of flow velocities of O_2^+ and O^+. The flow velocities for O_2^+ ($\mathbf{V}_{O_2^+}$; solid) and O^+ ($\mathbf{V}_{O^+}$; dashed) are derived from the MAVEN STATIC d1 data product (d0 used if d1 unavailable) and are corrected for the spacecraft velocity and spacecraft potential. The ion distributions are mostly broad in angular distributions, as shown in Supplementary Figs.Supplementary Fig. S1k and S1l, and clear deflections from the ram direction are observed in the instrument ϕ direction (Supplementary Fig. S1k). In other words, the STATIC instrument measures the majority of the ion distributions for this case study, and, thus, these measurements are appropriate to derive the ion bulk velocities. We have visually checked the ion angular distributions for other events to ensure that the calculation of the ion bulk velocity is appropriate. The uncertainty in the ion bulk velocity is mainly from the counting statistical uncertainty, which is included as error bars in Supplementary Fig. S1i and is shown in Supplementary Fig. S1j.

Zoomed-in views of MAVEN observations on 25 February 2017. Supplementary Figs. S3 and S4 show the zoomed-in views of the MAVEN observations for the two time periods of electron acceleration, respectively. Both figures show some rapid variations in the magnetic perturbation (Supplementary Fig. S3c) and thus the calculated $j^b_{//}$ (Supplementary Fig. S3e).

Additional case examples of MAVEN observations. Supplementary Fig. S5 to Supplementary Fig. S10 show the additional case examples of the MAVEN observations, in the same format as Supplementary Fig. S1. The dashed lines show the time intervals that are plotted in Fig. 4 in the main article.

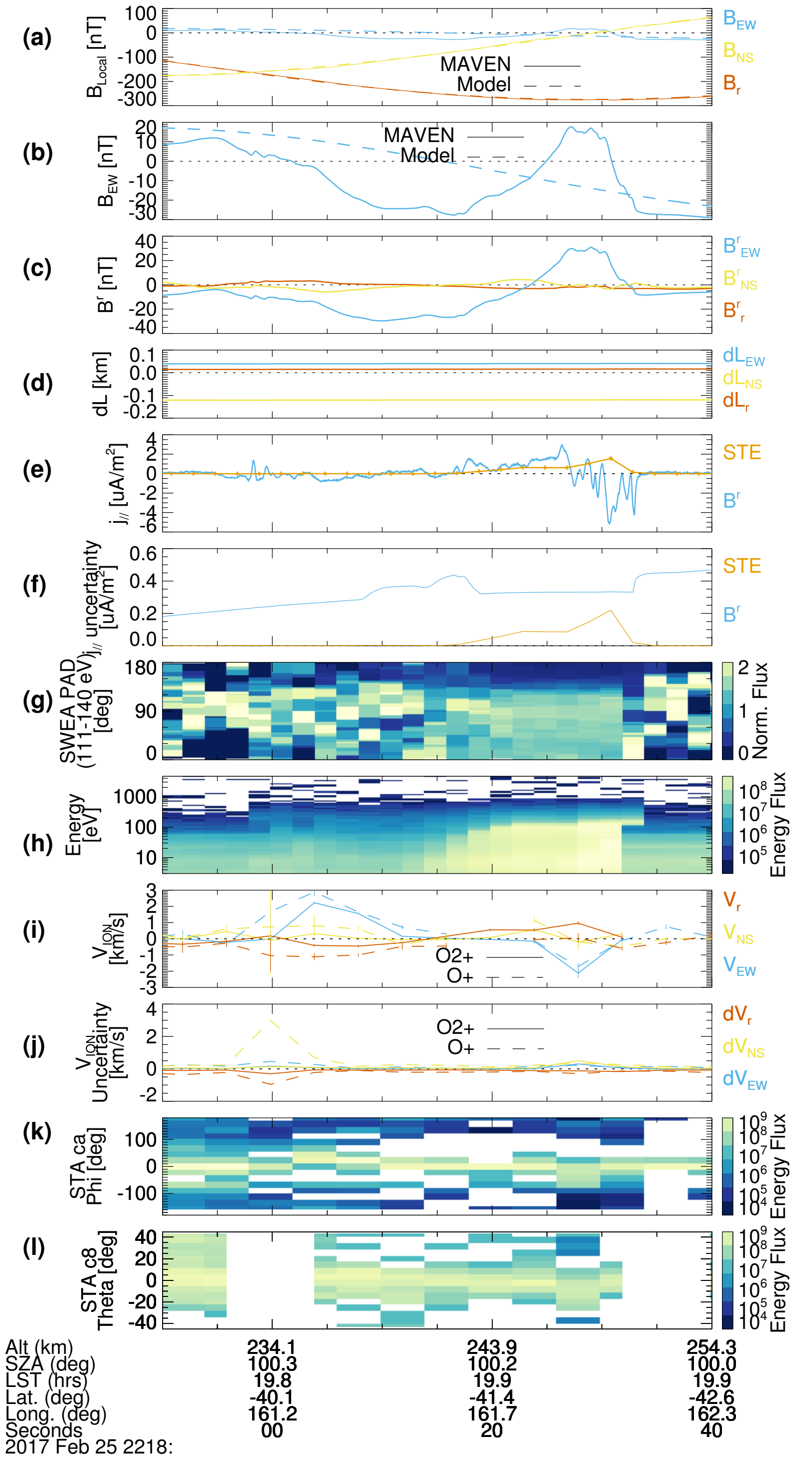

Supplementary Fig. S3 A zoomed-in view of MAVEN observations at 22:18 UT on 25 February 2017. A zoomed-in view of MAVEN observations at around 22:18 UT with the same format as Supplementary Fig. S1.

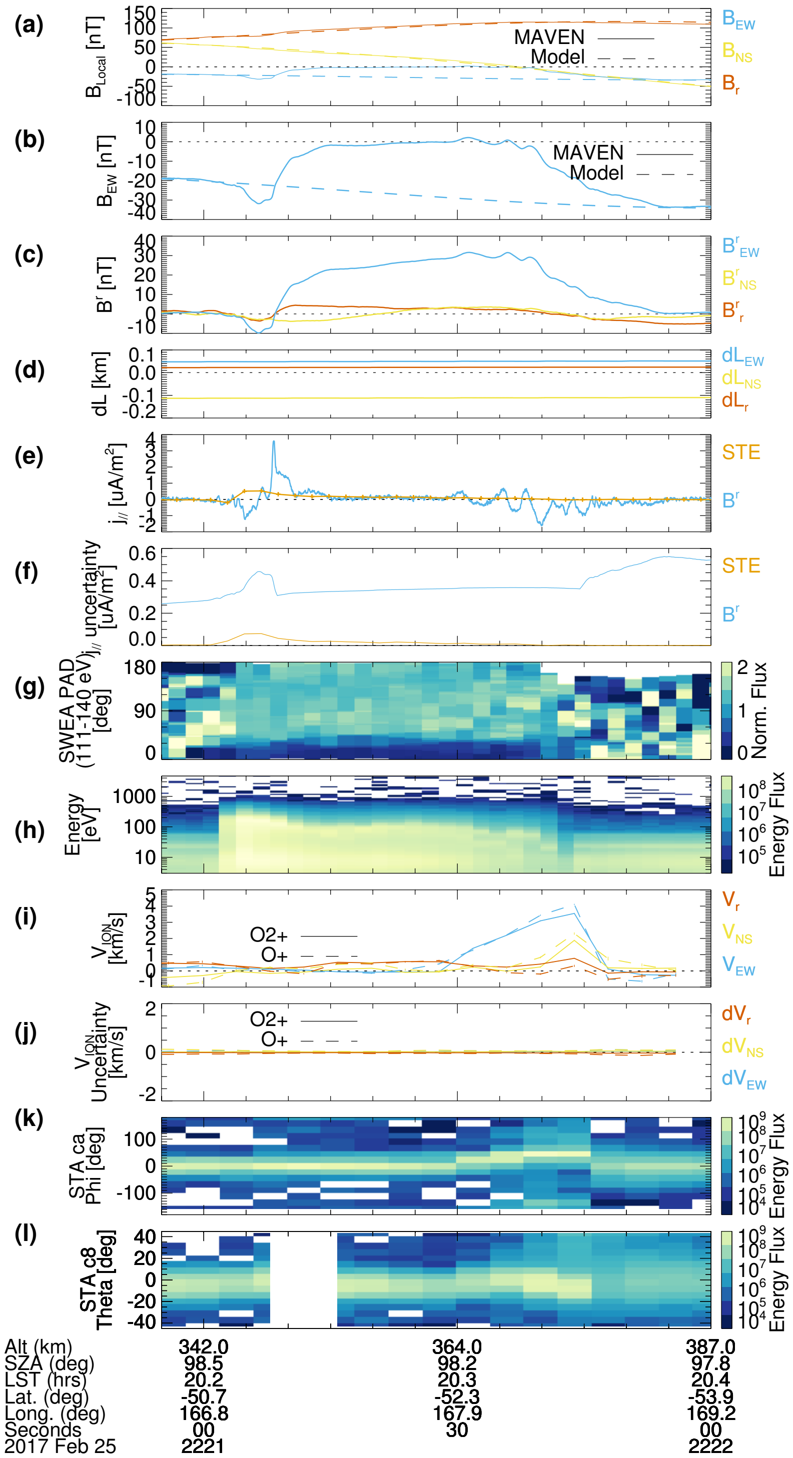

Supplementary Fig. S4 A zoomed-in view of MAVEN observations at 22:21 UT on 25 February 2017. A zoomed-in view of MAVEN observations at 22:21 UT with the same format as Supplementary Fig. S1.

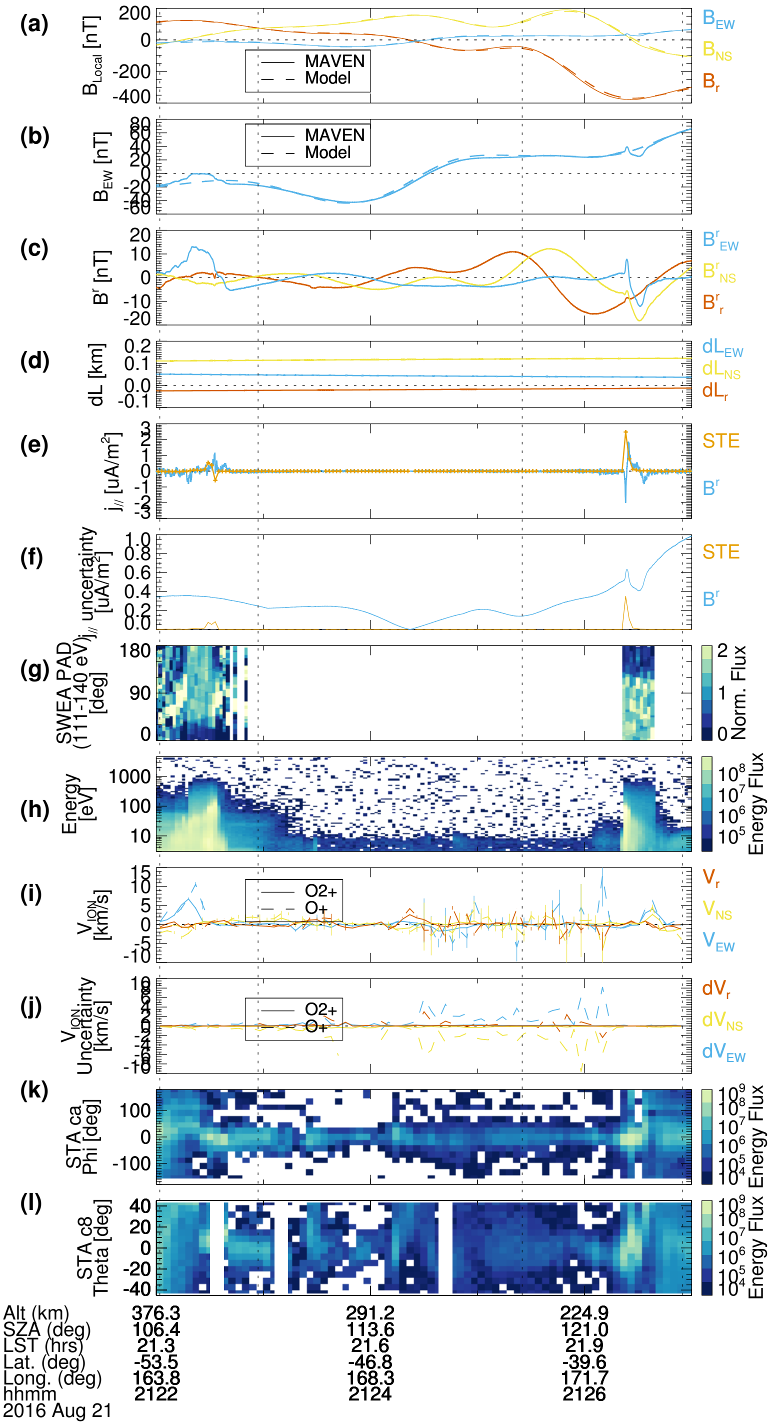

Supplementary Fig. S5 Additional MAVEN observations at 22 UT on 21 August 2016 (Event 1). Additional MAVEN observations at 22 UT on 21 August 2016 with the same format as Supplementary Fig. S1. The dashed lines show the time intervals that are plotted in Supplementary Fig.Figure 4 in the main article.

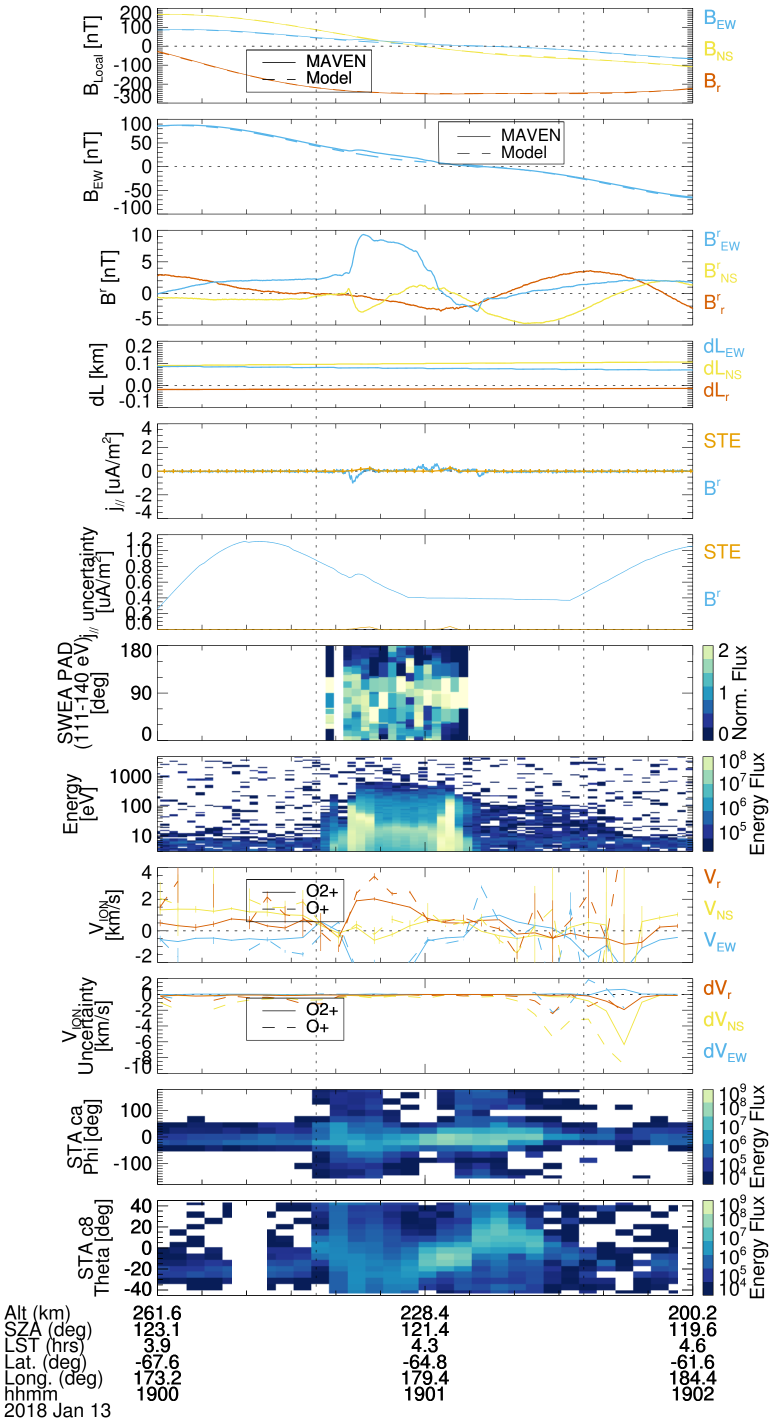

Supplementary Fig. S6 Additional MAVEN observations at 19 UT on 13 January 2018 (Event 3). Additional MAVEN observations at 19 UT on 13 January 2018 with the same format as Supplementary Fig. S1. The dashed lines show the time intervals that are plotted in Supplementary Fig.Figure 4 in the main article.

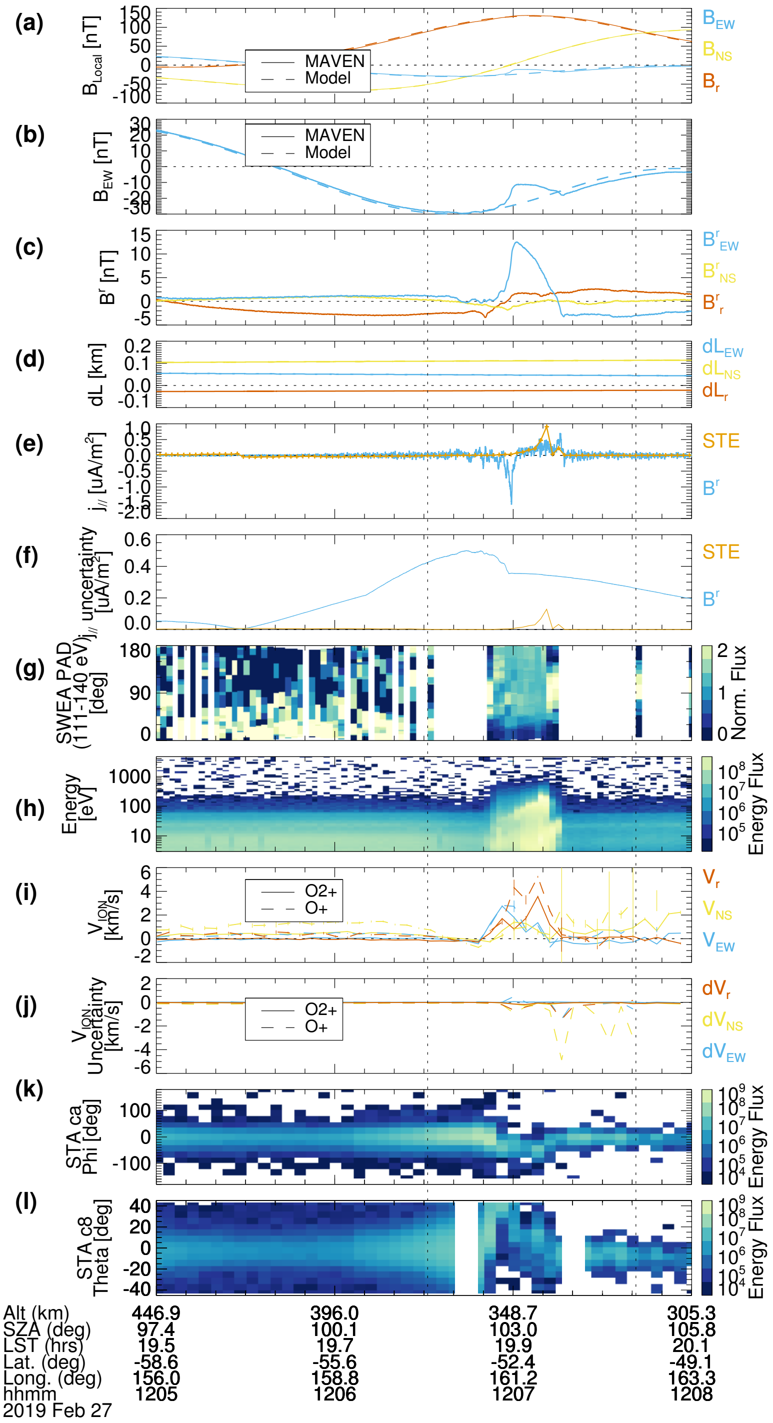

Supplementary Fig. S7 Additional MAVEN observations at 12 UT on 27 February 2019 (Event 4). Additional MAVEN observations at 12 UT on 27 February 2019 with the same format as Supplementary Fig. S1. The dashed lines show the time intervals that are plotted in Supplementary Fig.Figure 4 in the main article.

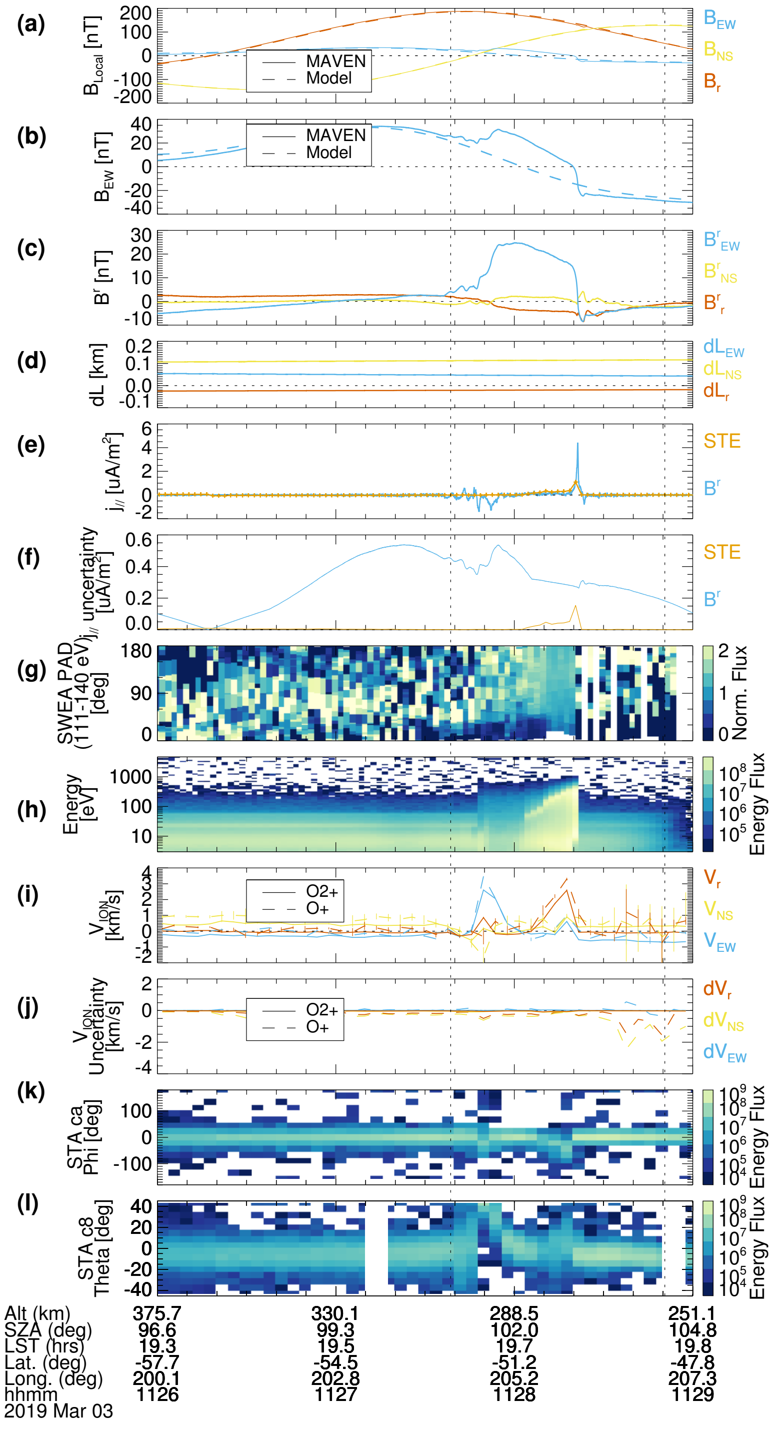

Supplementary Fig. S8 Additional MAVEN observations at 11 UT on 3 March 2019 (Event 5). Additional MAVEN observations at 11 UT on 3 March 2019 with the same format as Supplementary Fig. S1. The dashed lines show the time intervals that are plotted in Figure 4 in the main article.

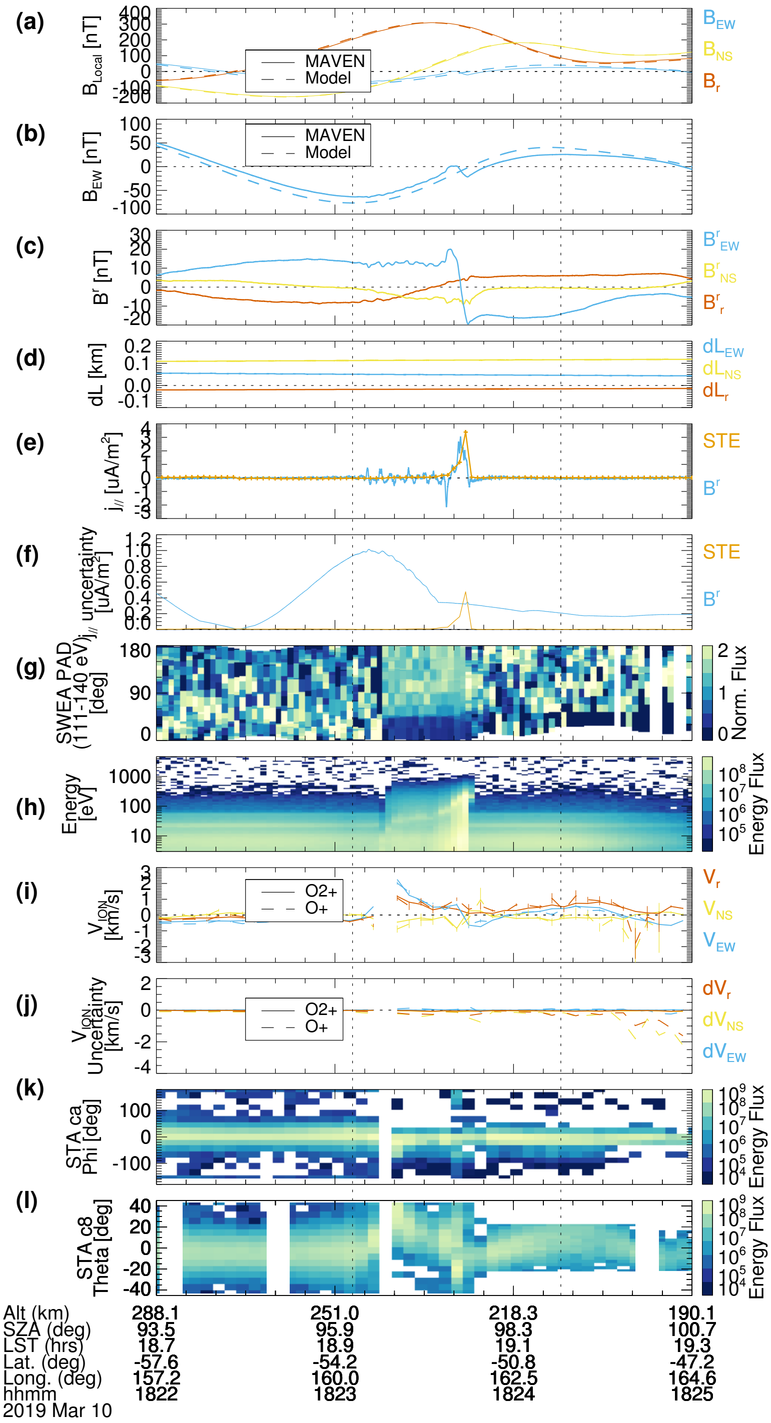

Supplementary Fig. S9 Additional MAVEN observations at 18 UT on 10 March 2019 (Event 6). Additional MAVEN observations at 18 UT on 10 March 2019 with the same format as Supplementary Fig. S1. The dashed lines show the time intervals that are plotted in Figure 4 in the main article.

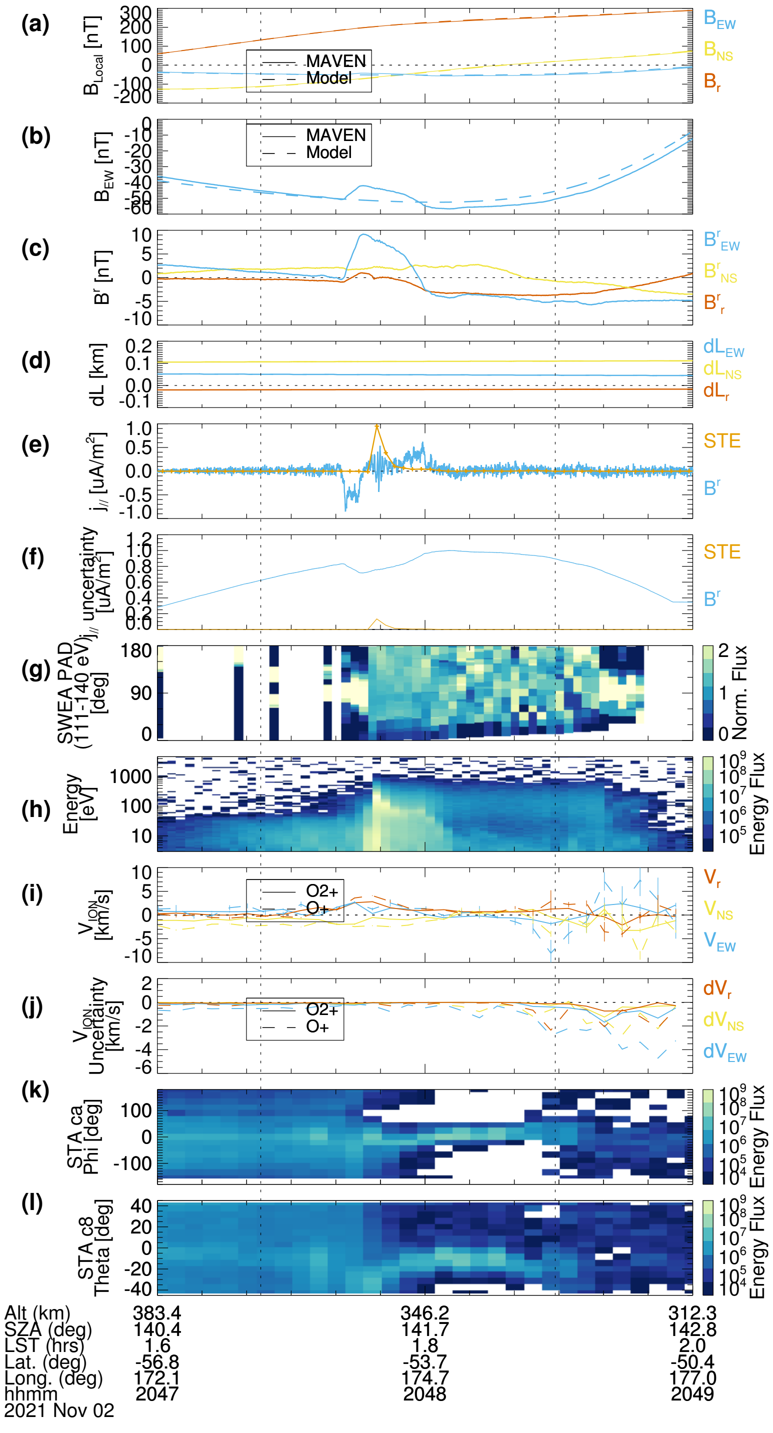

Supplementary Fig. S10 Additional MAVEN observations at 20 UT on 2 November 2021 (Event 7). Additional MAVEN observations at 20 UT on 2 November 2021 with the same format as Supplementary Fig. S1. The dashed lines show the time intervals that are plotted in Figure 4 in the main article.

References

[1] Langlais, B., Thébault, E., Houliez, A., Purucker, M. E. & Lillis, R. J. A new model of the crustal magnetic field of Mars using MGS and MAVEN. *Journal of Geophysical Research: Planets* **124**, 1542–1569 (2019).
