# Supplementary material for: Miniature Dungey-like cycle at Mars
Source: Nat Commun. 2026 Jul 23;17:6129. doi: 10.1038/s41467-026-75019-3 (PMC13396348; doi:10.1038/s41467-026-75019-3)
Supplement: Supplementary file 3 — Source data [file 41467_2026_75019_MOESM3_ESM.zip › data_files/README_SourceDataFile.rtf]

Source Data file for Miniature Dungey-like cycle at Mars.This zip file contains ASCII text files that contain the source plotting data, for each panel within each figure in the manuscript. Source data are provided for Figures 1, 4 and the Supplementary Figures 1-10. The naming convention of each ASCII file contains the date and time by the quantity contained within the file. There are two types of data files: (1) x-axis values and y-axis values and (2) x-axis values, y-axis values, and z-axis values. For Type-1 files (file names end with “B_EW, “B^r”, “Bcrust_model_local”, “Bmaven_local”, “dL”, “_eph”, “j_b_unc”, “j_b”, “j_ste_unc”,”j_ste”, “vel_o_GEL_local”,  “vel_o_unc_GEL_local”, “vel_o2_GEL_local”,  and “vel_o2_unc_GEL_local”): the first column is the x-axis value (the UTC timestamp); the second to last columns (if more than 2 columns) are the y-axis values. For Type-2 files (file names end with “mvn_sta_c8_Deflecter”, “mvn_sta_ca_Anode”, “mvn_swe_pad”, and “mvn_swe_spec”): the fourth row contains a list of y-axis values; the fifth row onward: the first column is the x-axis value (the UTC timestamp); the second to last columns are the z-axis values. The file names used for each panel in explained as follows:Figure 1Panel aFig_maven_20170225_221700_B^r.txtPanel bFig_maven_20170225_221700_j_ste.txtFig_maven_20170225_221700_j_b.txtPanel cFig_maven_20170225_221700_mvn_swe_spec.txtPanel dFig_maven_20170225_221700_vel_o_GEO_local.txtFig_maven_20170225_221700_vel_o2_GEO_local.txtFig_maven_20170225_221700_vel_o_unc_GEO_local.txtFig_maven_20170225_221700_vel_o2_unc_GEO_local.txtPanel eFig_maven_20170225_221700_j_ste.txtFig_maven_20170225_221700_j_b.txtFig_maven_20170225_221700_vel_o_GEO_local.txtFig_maven_20170225_221700_vel_o_GEO_local.txtFig_maven_20170225_221700_eph.txtFigure 4*All dates usedFig_maven_*_j_ste.txtFig_maven_*_j_b.txtFig_maven_*_vel_o_GEO_local.txtFig_maven_*_vel_o_GEO_local.txtFig_maven_*_eph.txtSupplementary Figures 1, 2-10*data of the date used for each figure is specified in the captionPanel aFig_maven_*_Bmaven_local.txtFig_maven_*_Bcrust_model_local.txtPanel bFig_maven_*_B_EWPanel cFig_maven_*_B^r.txtPanel dFig_maven_*_dL.txtPanel eFig_maven_*_j_ste.txtFig_maven_*_j_b.txtPanel_fFig_maven_*_j_ste_unc.txtFig_maven_*_j_b_unc.txtPanel_gFig_maven_*_mvn_swe_pad.txtPanel_hFig_maven_*_mvn_swe_spec.txtPanel iFig_maven_*_vel_o_GEO_local.txtFig_maven_*_vel_o2_GEO_local.txtPanel jFig_maven_*_vel_o_unc_GEO_local.txtFig_maven_*_vel_o2_unc_GEO_local.txtPanel kFig_maven_*_mvn_sta_c8_Deflecter.txtPanel lFig_maven_*_mvn_sta_ca_Anode.txtSupplementary Figure 2Fig_maven_20170225_221700_Bmaven_local.txtFig_maven_20170225_221700_Bcrust_model_local.txt
