# Supplementary material for: Miniature Dungey-like cycle at Mars
Source: Nat Commun. 2026 Jul 23;17:6129. doi: 10.1038/s41467-026-75019-3 (PMC13396348; doi:10.1038/s41467-026-75019-3)
Supplement: Supplementary file 2 — Transparent Peer Review file [file 41467_2026_75019_MOESM2_ESM.pdf]

nature portfolio

Peer Review File

Miniature Dungey-like cycle at Mars

Corresponding Author: Dr Shaosui Xu

This manuscript has been previously reviewed at another journal. This document only contains information relating to versions considered at Nature Communications.

This file contains all reviewer reports in order by version, followed by all author rebuttals in order by version.

Version 1:

Reviewer comments:

Reviewer #1

(Remarks to the Author)
The authors have appropriately addressed all of my comments on the previous submission, and I believe the revised manuscript is now suitable for publication in Nature Communications. I have one minor comment: although several auroral studies are discussed in the response to the reviewers, they are not cited in the manuscript. I suggest that these studies be discussed and the relevant references included in the manuscript as well.

Reviewer #2

(Remarks to the Author)
The authors have done an adequate job in revising the manuscript. However, I find that one critical aspect is still completely absent: the description of the error analysis and the derivation of the error bars need to be explained in detail. Without this information, it is not possible for readers or referees to evaluate the findings.

Reviewer #3

(Remarks to the Author)
All of my comments were reasonably addressed by the authors. I would recommend that the manuscript be publishable in the present form.

Version 2:

Reviewer comments:

Reviewer #1

(Remarks to the Author)
Thank you for addressing my comments and updating the manuscript. I believe the revised manuscript is suitable for publication. However, regarding the Code Availability statement, only a general SPEDAS link is provided. While the derived datasets are archived on Zenodo, I recommend that the code used for the analysis and visualization also be made available in a repository with a DOI, in accordance with the journal guidelines: https://www.nature.com/ncomms/editorial-policies/reporting-standards#replication-studies

Reviewer #2

(Remarks to the Author)
I find that the authors have addressed all comments satisfactorily.

Open Access This Peer Review File is licensed under a Creative Commons Attribution 4.0 International License, which permits use, sharing, adaptation, distribution and reproduction in any medium or format, as long as you give appropriate credit to the original author(s) and the source, provide a link to the Creative Commons license, and indicate if changes were made.
In cases where reviewers are anonymous, credit should be given to 'Anonymous Referee' and the source.
The images or other third party material in this Peer Review File are included in the article's Creative Commons license, unless indicated otherwise in a credit line to the material. If material is not included in the article's Creative Commons license and your intended use is not permitted by statutory regulation or exceeds the permitted use, you will need to obtain permission directly from the copyright holder.
To view a copy of this license, visit https://creativecommons.org/licenses/by/4.0/

Dear Editor and Reviewers,

We enclose the response to reviewers' comments in this document. We thank the reviewers for their efforts in reviewing the revised manuscript and for their further comments. We have addressed them in the response below and have changed the manuscript accordingly. The reviewers' comments are in **black**, and our responses are in **blue**.

We hope the editor and reviewers find the revised manuscript suitable for publication.

Sincerely,

Shaosui and Coauthors

Reviewer #1 (Remarks to the Author):

The authors have appropriately addressed all of my comments on the previous submission, and I believe the revised manuscript is now suitable for publication in Nature Communications. I have one minor comment: although several auroral studies are discussed in the response to the reviewers, they are not cited in the manuscript. I suggest that these studies be discussed and the relevant references included in the manuscript as well.

Right. The references not included in the previous manuscript are the discussion about the difference between the IUVS and EMUS aurorae. Initially, we did not include this discussion because we did not want to interrupt the flow of the paper too much. We have added this discussion in Section 3 in the revised manuscript (L295-313):

"It is also worth noting that the Emirates Ultraviolet Spectrometer (EMUS) [26] onboard the Emirates Hope Mars Mission (EMM) [27] has observed more diverse auroral forms than the MAVEN/IUVS observations at not only strong crustal fields but also at weakly magnetized regions [15, 28-30]. The EMM/EMUS instrument has a much higher sensitivity than the MAVEN/IUVS instrument. Consequently, EMUS can detect much weaker auroral emissions that result from the precipitation of solar wind electrons [15] and ionospheric photoelectrons [28, 30], and not just from energized or accelerated electrons that have much higher energy fluxes. In comparison, the auroral detections by the IUVS instrument are mostly limited to moderate/strong crustal field regions, suggesting that these emissions are triggered by electrons likely energized (with more intense fluxes) by processes associated with strong crustal fields. Another piece of supporting evidence is that the occurrence rate of EMUS aurorae is around 10%-30% (except for permanently closed crustal field regions) [28], comparable to the occurrence rates of magnetic field lines allowing for solar wind electrons or photoelectrons to precipitate onto the nightside atmosphere [31]. Meanwhile, the IUVS aurora is on the order of 0.1% to a few percent [13]. It suggests that aurorae detected by EMUS are sourced by common electron populations, while

aurorae detected by IUVS are sourced by comparatively rare electron populations, such as those accelerated by field-aligned potentials."

Reviewer #2 (Remarks to the Author):

The authors have done an adequate job in revising the manuscript. However, I find that one critical aspect is still completely absent: the description of the error analysis and the derivation of the error bars need to be explained in detail. Without this information, it is not possible for readers or referees to evaluate the findings.

We have provided descriptions of how we derive the uncertainties in the Supplementary Information (SI), but did not explicitly mention this information in the main manuscript. We have added the following sentence in the revised main article to highlight this: "The detailed explanations of the uncertainty calculations for $j^b_{//}$ and other key parameters are also provided in Supplementary Information." (L154-155)

Here is the information provided in the SI:
"The uncertainty in $j^b_{//}$ is calculated using the uncertainty in the measured magnetic field ΔB for each magnetic field component, where $\Delta B/|B| = 0.5\%$ for $|B| > 20$ nT and $\Delta B = 0.1$ nT for $|B| < 20$ nT." (L174-176 in SI)

"The uncertainty in $j^e_{//}$is calculated using the uncertainty in the measured electron flux, comprised of the counting statistic uncertainty, 10% uncertainty in the angular calibration, and 10% uncertainty in the absolute flux (mostly dominated by the last two terms)." (L190-193 in SI)

"The uncertainty in the ion bulk velocity is mainly from the counting statistical uncertainty, which is included as error bars in Figure S1i and is shown in Figure S1j." (L203-205 in SI)

Reviewer #3 (Remarks to the Author):

All of my comments were reasonably addressed by the authors. I would recommend that the manuscript be publishable in the present form.

Thanks!

Reviewer #1 (Remarks to the Author):

Thank you for addressing my comments and updating the manuscript. I believe the revised manuscript is suitable for publication. However, regarding the Code Availability statement, only a general SPEDAS link is provided. While the derived datasets are archived on Zenodo, I recommend that the code used for the analysis and visualization also be made available in a repository with a DOI, in accordance with the journal guidelines: https://www.nature.com/ncomms/editorial-policies/reporting-standards#replication-studies

We have revised our Code Availability section following a recently published NC paper* that is analyzed in the same fashion using MAVEN data:
"Data access and processing were done using SPEDAS V3.1 (http://spedas.org/wiki/index.php?title=Downloads_and_Installation) [35]. Code written to perform the detailed analysis and to produce the figures in this manuscript can be provided upon request, but with no guarantee that it will work on individual user systems and setups."

*Fowler, C.M., Hanley, K.G., McFadden, J. et al. Detection of Zwan-Wolf effect in the ionosphere of Mars. Nat Commun 17, 4224 (2026). https://doi.org/10.1038/s41467-026-72251-9
